# ESPO and Health Sciences Section Symposium: A Multidimensional View of Aging: Utilizing Innovative Approaches in Gerontology

**DOI:** 10.1093/geroni/igaf122.1253

**Published:** 2025-12-31

**Authors:** Kate Perepezko, Tingzhong (Michelle) Xue, Hanzhang Xu

## Abstract

As our aging population grows, understanding the aging process is increasingly critical for developing strategies to enhance the health and quality of life of our population. Aging is a highly individualized process shaped by biological, interpersonal, and social factors. Therefore, addressing the complexities of aging requires innovative methodological and analytical approaches to explore unanswered questions in gerontology. This symposium will showcase cutting-edge strategies for studying key issues affecting the aging population, emphasizing innovative research methods and interdisciplinary collaboration. This session highlights the work of early career scientists who utilize advanced methodological and analytical approaches to explore key topics in gerontology. Dr. Kate Singer will present her work exploring how recalling stressful caregiving experiences affects caregiver stress levels and cognitive performance using functional near-infrared spectroscopy (fNIRS). Drs. Hyojin Choi and Kate Perepezko will present their work examining the interdependence of frailty among caregiving dyads, comparing homebound dyads to non-homebound dyads. Dr. Tingzhong Xue will present a study aimed to examine the roles of behavioral symptoms and vision loss on physical resilience among nursing home residents with dementia after hip fracture. Jiameng Yuan will present her evaluation of the implementation of a telemedicine program, EAGLEcareACT (Enhancing Advance Care Planning, Geriatrics, and End-of-Life Care Acute Care Team) in Singapore. This symposium will conclude with a discussion led by Dr. Hanzhang Xu who will synthesize key insights from these presentations, exploring common methodological advancements, their implications for intervention design, and future directions for aging research.

